# Biofortification: Future Challenges for a Newly Emerging Technology to Improve Nutrition Security Sustainably

**DOI:** 10.1016/j.cdnut.2024.104478

**Published:** 2024-10-19

**Authors:** Howarth Bouis, Jennifer Foley, Keith Lividini, Jaya Jumrani, Russell Reinke, Dominique Van Der Straeten, Ronan Zagado, Erick Boy, Lynn R Brown, Bho Mudyahoto, Richard Alioma, Munawar Hussain, Wolfgang H Pfeiffer

**Affiliations:** 1 International Food Policy Research Institute, Washington, DC; 2 HarvestPlus, International Food Policy Research Institute, Washington, DC; 3 Micronutrient Forum, Washington, DC; 4 ICAR - National Institute of Agricultural Economics and Policy Research (NIAP), New Delhi, India; 5 International Rice Research Institute, Los Baños, Laguna, Philippines; 6 Laboratory of Functional Plant Biology, Ghent University, B-9000 Ghent, Belgium; 7 Philippine Rice Research Institute, Muñoz, Nueva Ecija, Philippines; 8 Alliance Bioversity & CIAT, Rome, Italy

**Keywords:** biofortification, malnutrition, micronutrients, nutrition security, cost-effectiveness, food staples, agricultural research

## Abstract

Biofortification was coined as a term to define a plant breeding strategy to increase the micronutrient content of staple food crops to reduce the burden of micronutrient deficiencies in low- and middle-income countries. In 2003, the HarvestPlus program, based in the centers comprising the Consultative Group on International Agricultural Research, was initiated to implement the biofortification strategy. This article discusses what has been achieved, what has been learned, and the key challenges to embed biofortification in food systems and to expand its impact. Cost-effectiveness is key to the biofortification strategy. Biofortification piggybacks on the agronomically superior varieties being developed at agricultural research centers. Central plant breeding research discoveries can be spread globally. Farmers have every motivation to adopt the latest high-yielding, high profit crops. High productivity leads to lower food prices. As a consequence, consumers can increase their mineral and vitamin intakes at no additional cost by substituting biofortified staple foods 1-for-1 for nonbiofortified staple foods. After 20 years of investment, biofortified staple food crops are being produced by farmers in over 40 countries and are eaten by hundreds of millions of people. Published nutrition trials have shown nutrient-rich crops to be efficacious. The biofortification strategy is now recognized by the international nutrition community as one effective approach among several interventions needed to reduce micronutrient deficiencies. This is a promising beginning. However, biofortification is still a newly emerging technology. A limitation of biofortification as implemented to date is that densities of single nutrients have been increased in given staple food crops. To reach a higher trajectory, the impacts of biofortification can be multiplied several-fold using genetic engineering and other advanced crop development techniques to combine multiple-nutrient densities with climate-smart traits.

## Introduction

Biofortification was coined as a term to define a plant breeding strategy to increase the micronutrient content of staple food crops to reduce the burden of micronutrient deficiencies in low- and middle-income countries (LMICs). The potential contribution of more nutrient-dense food staples to improving public health was first addressed formally by the centers comprising the Consultative Group on International Agricultural Research (CGIAR) in 1994 at a meeting convened by the International Food Policy Research Institute (IFPRI). Nine years later, in 2003, the HarvestPlus “biofortification” program was initiated within CGIAR. In the intervening 20 years, HarvestPlus has invested ∼USD $500 million in developing, testing, and scaling up biofortified varieties of 12 staple food crops that are now are now enriching the diets of hundreds of millions of people. (HarvestPlus has invested primarily in zinc rice, zinc wheat, provitamin A maize, provitamin A cassava, iron beans, and iron pearl millet. Initially provitamin A sweet potato was included under the HarvestPlus umbrella, but in 2010 spun off under the direction of the International Potato Center. Smaller investments were made by HarvestPlus in provitamin A banana-plantains, iron and zinc cowpea, lentils, potato, sorghum, and zinc maize. Biofortified potato varieties have not yet been released).

Since 1960, CGIAR has been successfully developing and disseminating **high yielding** varieties of wheat and rice in South and Southeast Asia, among other food crop technologies, which are attractive to farmers globally. Today, these CGIAR technologies have been adopted on over 221 million hectares of land, and they generate food eaten ≤3 times a day, by poor and rich consumers alike across Asia, Africa, and Latin America [1].

In retrospect, to have simultaneously increased the density of nutrients such as iron and zinc in these varieties (to have “biofortified” these crops) alongside improving their agronomic performance would have required a one-time extra cost in the breeding effort. Therein lies the comparative advantage of biofortification: the investment generates long-term nutritional and economic benefits that accrue year after year; the plants do the work.

Breeding for the characteristic of nutrient density piggybacks upon existing and ongoing efforts to develop and release crop varieties with increased yield, improved disease and pest resistance, resilience to climate change, and end-use quality requirements. Nutritional traits in improved varieties remain stable over time, fixed in the germplasm. This is unlike supplementation and fortification, which incur repeated annual costs. Biofortified varieties developed at a central location can be spread globally (especially, and importantly, to rural populations) and adapted to local growing conditions. Biofortified foods sell for the same price as conventional local varieties due to their competitive yields with no change in production costs.

The added difficulty with adding provitamin A density is that consumers must accept a color change in their staple foods from white to yellow or orange (due to the added carotenoids). There is an added cost of creating a permanent demand for this visible trait based on the value proposition that for the same price as nonbiofortified varieties, biofortified varieties can protect individuals and their families from vitamin A deficiencies.

In a nutshell, this is the concept behind biofortification.

### Key questions

For biofortification to be successful, however, it was necessary over time that each one of the following questions be answered positively:

• Is biofortification more cost-effective than existing interventions?
• Would the strategy provide enough micronutrients with sufficient bioavailability to have a public health impact?
• Could high nutrient density be combined with high yield and high farmer profits?
• Would farmers adopt the new varieties and would consumers accept them and demand them, especially where there is a change in color from white to yellow or orange?
• Could donor and policymaker interest be sustained over the 4 decades required to “mainstream” biofortification in food systems?

This article provides a comprehensive update on the progress achieved by collaborating institutions implementing biofortification under the CGIAR-sponsored HarvestPlus program and addresses some criticisms of the strategy. The review discusses the cost-effectiveness of biofortification, its impact on human nutritional status and health, the process of breeding for nutrient density and varietal release, the adoption of the technology by farmers and consumers, policies linking agriculture and nutrition, and challenges facing the wide-scale implementation of biofortification moving forward.

### Cost-effectiveness

**FIGURE 1:**
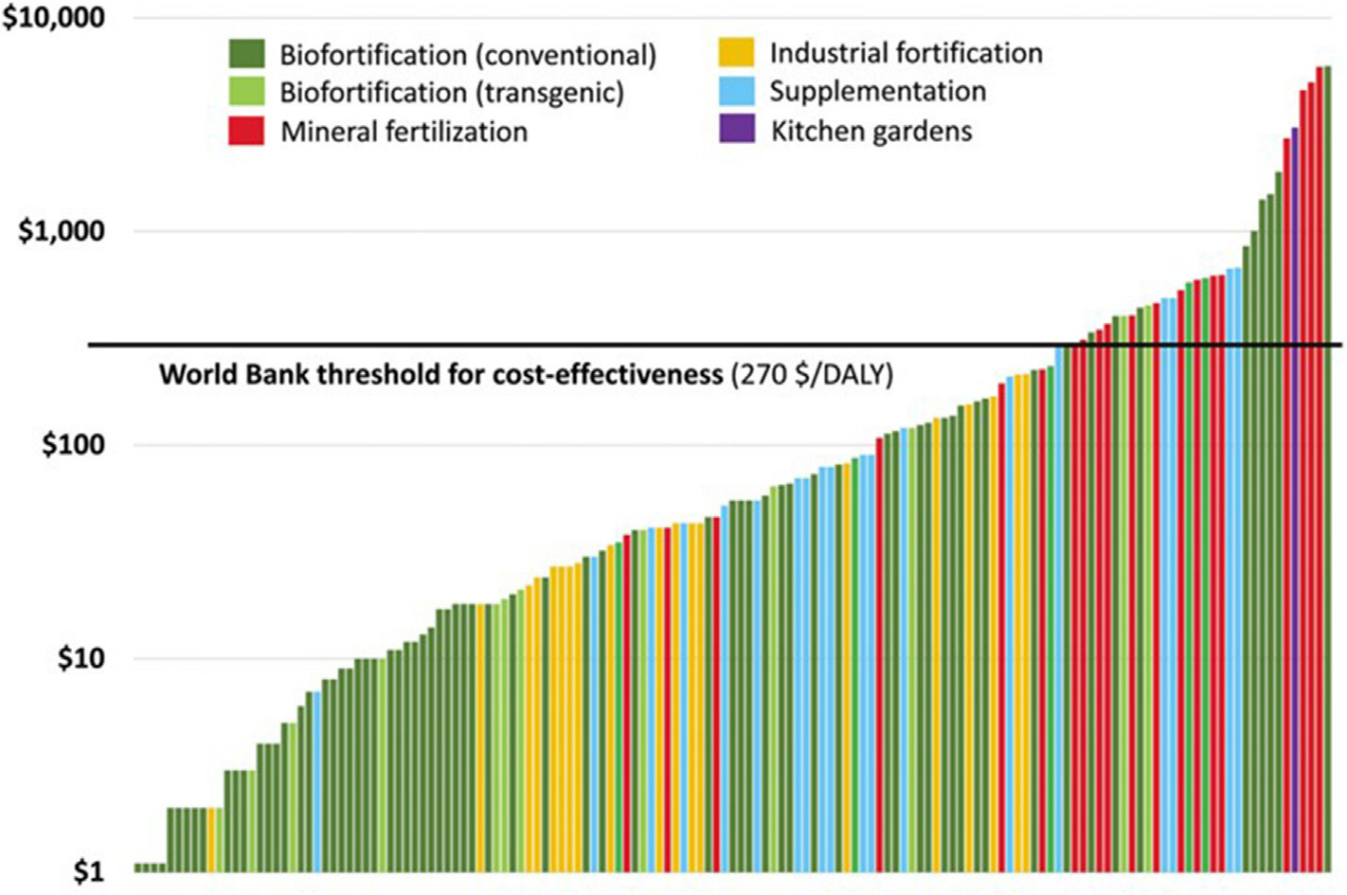
Cost per disability adjusted life year (DALY) saved for a range of micronutrient interventions. Cost-effectiveness of different micronutrient interventions: Compiled by Matin Qaim and Alexander Stein based on a large number of original cost-effectiveness studies identified through a systematic literature review. Each column represents one cost-effectiveness estimate expressed in terms of the cost in USD per DALY saved (log scale). (Reproduced with permission from reference [1].)

To address the first question raised above, several ex ante benefit-cost studies of biofortification have been undertaken to justify pursuing biofortification on the basis of its cost-effectiveness. As shown in Figure 1, the cost per disability-adjusted life year saved is lowest for the green shaded biofortification interventions [1]. This indicates high **potential** economic return: **if the varieties are widely adopted**, these benefits accrue year after year at very little marginal cost.

The added nutrient density, the average consumption of the biofortified food, percentage replacement of the nonbiofortified varieties with biofortified varieties, and initial disease burden are the main factors driving the cost-effectiveness of biofortification programs. Biofortification interventions tend to target rural areas initially due to their high consumption of staples from home production [[2], [3], [4]]. Ex post cost-effectiveness analyses of biofortified orange sweet potato in rural areas in 2 countries have also shown that biofortification is highly cost-effective [5,6].

Additional reviews and summaries covering the ex ante cost-effectiveness of biofortification conclude that biofortification has the potential to be a highly cost-effective intervention under many scenarios [[7], [8], [9]]. Lividini et al. [7] found that roughly 75% of pessimistic scenarios for biofortification (which assumed slower and lower-than-maximum scale adoption rates and lower additional micronutrient densities as compared with optimistic scenarios) still achieved the threshold value for high cost-effectiveness. In studies by Meenakshi et al. [10], Chow et al. [11], and Stein [12] countries with relatively lower values for these same conditions were typically the least cost-effective [7].

Importantly, where alternative micronutrient interventions have been compared within the same country, biofortification has been shown to be cost-effective, competitive [2,4,[13], [14], [15]], and part of an effective package of complementary interventions [4,13,14]. Biofortification has also been found to be cost-effective relative to other health interventions [16]. Resilience is an additional benefit as shown by the expansion of biofortification during major food systems disruptions, such as the COVID-19 pandemic (see example below of zinc wheat in Pakistan).

The Fill the Nutrient Gap research [17] has illustrated the impact that biofortification can have on the cost of a nutritionally adequate diet. Analysis by the World Food Programme showed that in the Punjab province of Pakistan, household access to biofortified zinc wheat would result in a 12.6% and 11.5% reduction in rural and urban costs of a nutritious diet, respectively; results indicated the cost of a nutritious diet for an adolescent girl in Punjab would fall by 26% in rural areas and by 25% in urban areas [18]. Given norms in Pakistan, where women and girls often have less access to preferred foods, including animal source foods, and eat last and least in the household, incorporation of nutrients in staple grains can have substantial and equitable impacts [[19], [20], [21], [22]]. Drivers of this result are that wheat is the primary staple eaten every day, zinc wheat sells for the same price as nonbiofortified wheat (see Supplementary Material 1), women and girls are more likely to eat proportionately more grains in the household, and zinc wheat also has higher iron content compared to other conventional wheat varieties, for which adolescent girls have relatively higher physiological requirements.

Finally, although it continues to face political and regulatory hurdles, transgenic biofortification offers great potential for even higher cost-effectiveness due to the ability to more quickly include **multiple** high nutrient density traits within single biofortified varieties using gene stacking [1,8,9].

## Understanding How the Role of Food Staples in LMIC Diets Drives the Benefits of Biofortification for the Poor

Having presented the evidence on cost-effectiveness, it is useful to describe and understand the pattern of food staple intakes in LMICs and their current contribution to mineral and vitamin intakes that drive these high economic returns. First, consumers across the income spectrum within any given LMIC typically eat about the same amount of food staples [[23], [24], [25]]. Second, food staples provide a very significant base intake of minerals and vitamins, which predominate more in the diets of low-income consumers than those of high-income consumers.

### Intakes of food staples

Food staples are the least expensive source of energy intake and so are eaten in large quantities to avoid hunger. Consumers at all income levels within a given country typically eat about the same amount of food staples. As incomes increase, consumers can afford to diversify their diets by consuming larger amounts of highly desired vegetables, fruits, pulses, and animal and fish products. Using household-level food intake data expressed in per capita terms, this pattern was recognized 30 years ago [[23], [24], [25]].

The same pattern is observed in dietary intake data collected from individual adult women and preschoolers in poor rural areas in 7 countries (Table 1 [[26], [27], [28], [29], [30], [31], [32], [33], [34], [35]], Supplementary Table 1). Staple food consumption is nearly constant across wealth terciles. (Further analysis is ongoing to evaluate factors driving intrahousehold consumption by sex and age.) Percentage increases across wealth terciles are generally highest for animal and fish products, although starting from low bases. The percentage of total calories provided by staple foods is highest for the poorest tercile. In periods of crisis when food prices increase, it is staple food intake that consumers protect, and adjustments are made to food purchases by reducing the quantities eaten of nonstaple foods [36,37].

**TABLE 1:** Mean energy intakes (kilocalories per day) from different food groups among mothers and their children, by wealth category^1^^,^^2^.

	Mothers					Children				
	Poorest	Less poor	Least poor	All	Least poor/ poorest	Poorest	Less poor	Least poor	All	Least poor/ poorest
Staple foods										
Nigeria	1500	1519	1514	1511	0.9%	626	619	663	635	5.9%
Burkina Faso	1945	1800	1733	1826	−10.9%	1063	1027	986	1025	−7.2%
Zambia	1954	1974	1762	1899	−9.8%	1257	1176	1193	1212	−5.1%
Mozambique Baseline	2064	2029	2117	2069	2.6%	761	703	763	743	0.2%
Mozambique Endline	1818	1801	1804	1808	−0.8%	966	1082	1001	1016	3.6%
Uganda Baseline	1851	2082	2159	2031	16.7%	1186	1295	1357	1280	14.3%
Uganda Endline	1775	1829	1748	1784	−1.5%	1346	1335	1329	1337	−1.2%
Philippines	1856	1831	1797	1830	−3.2%	948	910	929	930	−2.0%
Bangladesh	1918	2008	1953	1960	1.8%	980	914	894	937	−8.8%
Fruits, vegetables, pulses										
Nigeria	175	197	204	191	16.9%	94	99	85	93	−9.5%
Burkina Faso	79	80	91	84	15.2%	52	55	65	58	25.0%
Zambia	82	88	106	91	29.3%	64	60	79	68	23.0%
Mozambique Baseline	49	56	65	57	32.9%	33	35	43	37	32.9%
Mozambique Endline	45	59	68	57	50.4%	37	45	50	44	35.3%
Uganda Baseline	168	225	273	222	62.6%	133	173	216	174	62.8%
Uganda Endline	182	266	365	271	101.1%	163	246	326	245	100.0%
Philippines	92	108	112	103	20.7%	47	57	60	54	28.1%
Bangladesh	211	258	265	245	25.3%	109	112	137	117	25.3%
Animal and fish foods										
Nigeria	306	359	372	346	21.7%	144	165	176	162	22.8%
Burkina Faso	23	39	46	36	100.0%	16	30	29	25	81.3%
Zambia	53	74	165	95	209.7%	40	53	103	64	155.4%
Mozambique Baseline	70	137	109	105	56.2%	40	53	53	49	32.3%
Mozambique Endline	64	87	87	80	35.4%	48	59	60	56	24.8%
Uganda Baseline	117	115	163	132	39.9%	100	89	115	101	14.7%
Uganda Endline	145	187	237	190	63.1%	86	116	151	118	75.6%
Philippines	84	103	205	128	144.7%	61	65	140	85	131.4%
Bangladesh	41	59	82	61	99.2%	33	43	70	46	111.8%

1 A more disaggregated enumeration of food intakes and description of the data sets used is provided in Supplementary Table 1.

2 Food intake data were collected using a 24-h recall methodology. The samples are not nationally representative and were all conducted in purposively identified rural areas. The Nigeria [26], Burkina Faso [27], Zambia [28], Mozambique [29,30,31], and Uganda [32,33] data sets were collected under the HarvestPlus program as described in Section 3 of the article. The Uganda and Mozambique baseline and endline surveys were conducted as panel surveys as part of an orange sweet potato effectiveness trial. The Philippines [34] and Bangladesh [35] data sets are also panel surveys over four rounds. For the Philippines and Bangladesh data sets the number of observations counts the same women and children multiple times.

### Mineral and vitamin density in food staples multiplied by intake

A common refrain is that “food staples lack minerals and vitamins.” A more precise, accurate statement is that “food staples are not as dense in mineral and vitamins relative to nonstaple foods.” Intake of minerals and vitamins from food staples is calculated as the bioavailable density times the edible quantity consumed. What is often overlooked is that food staples provide a substantial base intake of a wide range of minerals and vitamins.

This is shown in Table 2 [38], which is constructed from a nationally representative dietary survey for the Philippines. On average, milled rice provides a substantial intake share (above 30%) of iron, thiamine (vitamin B1), and niacin (vitamin B3), as well as vitamin B5, vitamin B6, zinc, copper, magnesium, manganese, phosphorus, and several amino acids. Since rice intakes do not vary with income and non-staple intake is lower for poor income groups, these percentages for rice for the lowest income groups are well above the national average (see Table 1 in [1]).

**TABLE 2:** Food group percentage contributions to total nutrient intakes, Philippines, 2015.

Nutrient/food group	Percent contribution to total intakes				
	Rice and rice products^1^	Energy-giving foods^2^	Body-building foods^3^	Body-regulating foods^4^	Miscellaneous^5^
Energy	56%	77%	17%	3%	3%
Protein	37%	48%	47%	4%	1%
Fats	4%	48%	50%	1%	1%
Carbohydrates	73%	91%	2%	4%	3%
Iron	30%	48%	30%	15%	7%
Calcium	19%	30%	46%	18%	6%
Thiamine	35%	59%	29%	9%	4%
Riboflavin	19%	34%	51%	11%	4%
Niacin	43%	52%	38%	4%	5%
Vitamin A	0%	7%	72%	17%	5%
Vitamin C	0%	8%	6%	77%	9%
Folate^6^	10%	40%	31%	27%	2%
Vitamin B5^6^	58%	76%	19%	4%	0%
Vitamin B6^6^	33%	49%	29%	20%	2%
Vitamin D^6^	0%	0%	100%	0%	0%
Vitamin E^6^	1%	45%	32%	15%	8%
Copper^6^	32%	56%	22%	12%	10%
Magnesium^6^	39%	67%	15%	12%	7%
Manganese^6^	54%	81%	10%	5%	3%
Phosphorus^6^	34%	59%	34%	5%	2%
Potassium^6^	15%	40%	27%	25%	9%
Zinc^6^	41%	60%	33%	5%	1%

Rice and rice products account for 30%–40% of total intakes for 10 of 11 amino acids.

HarvestPlus estimated densities for non-biofortified milled rice are 16 mg/kg Zn and 2 mg/kg Fe. For milled rice, the IML FCT value is 10.6 mg/kg Zn, and the Philippine FCT value is 10 mg/kg Fe. Thus, the zinc percentage for rice in the table above is likely understated, and the iron percentage is likely overstated. Additional detail by income group is provided in [1], Table 1.

1 Intake of rice products accounts for 1.5% of total rice and rice products intake as measured by energy.

2 Energy-giving = rice, maize, wheat, roots and tubers, sugar and syrups, fats and oils.

3 Body-building = animal and fish products; beans, nuts, and seeds.

4 Body-regulating = vegetables and fruits.

5 Miscellaneous = beverages, condiments and spices, others.

6 Unpublished analysis using the International Mini List (IML) Food Composition Table (FCT) values; values for these nutrients are not available in the Philippine FCT.

To investigate if data for other countries show the same pattern, we utilized data from nutrient balance sheets (NBS), which provide macro- and micronutrient balances for 34 nutrients through year 2018 [39]. Countries were grouped into 10 subregional typologies defined by primary food staples consumed. The percentage contribution of each staple (maize, wheat, rice, cassava, sorghum, millet, beans, potatoes, sweet potatoes) to total apparent per capita dietary energy intake was calculated and ranked for each country. Staples that provided ≥10% of total apparent per capita dietary energy intake were considered significant contributors to dietary energy intake. Countries were grouped by similar food staple consumption patterns with an objective of minimizing the total number of typologies (see Supplementary Table 2).

The percent contributions of each staple included in a given typology, to total apparent per capita micronutrient intake, and to population-weighted daily micronutrient requirements were calculated as the mean of the 3 most recent years included in the NBS (2016–2018) to smooth any annual outliers. Calculations for each country and each typology (weighted by population) are shown in Supplementary Table 2; a comparison for the Philippines of Table 2 and the NBS data are also presented in Supplementary Table 2.

The 10 typologies were then combined into 2 summary tables by region (Africa, Asia, and Latin America), which present the population-weighted regional averages of staples included in any of the regional typologies. Table 3 [39] presents the average percent contributions of each staple to total apparent per capita dietary energy and micronutrient intake. Table 4 [39,40] presents the average percent contributions of each staple to total population-weighted daily dietary energy and micronutrient requirements based on the Harmonized Average Requirements [40].

**TABLE 3:** Percent contribution of primary staples to total intake of selected nutrients by region.

Region	Asia				Africa							Latin America			
Top staples	Rice	Wheat	Maize	Top 3	Rice	Wheat	Maize	Cassava	Sorghum	Millet	Top 6	Rice	Wheat	Maize	Top 3
Percentage energy of staple	31%	18%	4%	54%	11%	8%	16%	11%	6%	4%	56%	7%	11%	13%	31%
Staple as a % of top 3 staples (Asia, LAC) & top 6 (Africa)	58%	34%	7%	100%	20%	15%	28%	20%	11%	6%	100%	23%	35%	42%	100%
Iron (Fe)	17%	26%	4%	47%	7%	7%	19%	4%	7%	4%	48%	5%	9%	12%	26%
Zinc (Zn)	29%	30%	4%	63%	12%	8%	22%	7%	5%	4%	58%	8%	9%	14%	31%
Thiamine (B1)	31%	22%	5%	59%	11%	7%	22%	10%	6%	5%	60%	9%	10%	14%	34%
Riboflavin (B2)	11%	14%	3%	28%	5%	4%	16%	9%	2%	5%	40%	2%	3%	5%	10%
Niacin (B3)	38%	27%	3%	69%	15%	6%	19%	9%	7%	5%	60%	12%	6%	10%	27%
Pantothenic acid (B5)	36%	20%	2%	58%	17%	6%	10%	4%	3%	4%	44%	11%	6%	4%	20%
Vitamin B6	34%	12%	7%	53%	12%	2%	26%	7%	5%	4%	55%	10%	2%	19%	30%
Folate (B9)	9%	14%	2%	25%	3%	6%	8%	13%	3%	4%	37%	2%	9%	9%	20%
Calcium (Ca)	13%	6%	0%	20%	5%	3%	2%	9%	1%	0%	21%	2%	2%	10%	15%
Copper (Cu)	26%	26%	3%	55%	10%	7%	15%	9%	3%	7%	51%	9%	11%	11%	31%
Potassium (K)	14%	15%	2%	31%	4%	3%	10%	14%	4%	2%	36%	3%	4%	9%	15%
Magnesium (Mg)	28%	25%	5%	58%	11%	5%	24%	8%	7%	5%	61%	10%	7%	18%	35%
Manganese (Mn)	38%	34%	1%	73%	21%	10%	8%	11%	6%	5%	62%	21%	13%	8%	42%
Sodium (Na)	4%	1%	3%	8%	2%	1%	14%	11%	0%	1%	28%	1%	0%	2%	3%
Phosphorus (P)	28%	25%	3%	56%	12%	7%	18%	5%	7%	5%	54%	8%	7%	14%	29%
Selenium (Se)	41%	23%	4%	68%	17%	18%	21%	2%	7%	1%	66%	10%	21%	11%	43%
Vitamin A	0%	0%	0%	0%	0%	0%	2%	1%	0%	0%	3%	0%	0%	0%	0%
Vitamin C	0%	0%	0%	0%	0%	0%	0%	28%	0%	0%	29%	0%	0%	0%	0%
Vitamin D	0%	0%	0%	0%	0%	0%	0%	0%	0%	0%	0%	0%	0%	0%	0%
Vitamin E	5%	8%	1%	14%	2%	1%	5%	4%	3%	0%	15%	1%	1%	2%	4%
Vitamin K	1%	2%	0%	3%	0%	0%	0%	4%	0%	0%	5%	0%	0%	0%	0%

Abbreviation: LAC, Latin American countries.

The contributions of 9 staple crops (maize, wheat, rice, cassava, sorghum, millet, beans, potatoes, sweet potatoes) to total apparent per capita dietary energy intake were computed by country. Multiple typologies of the greatest contributions of staples to dietary energy were constructed by region. Staples identified in the typologies across each region are shown in the table. From among the countries included in each region, the population-weighted average percent contributions of each staple to total apparent per capita dietary energy and micronutrient intake are shown.

**TABLE 4:** Percent contribution of primary staples to daily requirements of selected nutrients by region.

Region	Asia				Africa							Latin America			
Top staples	Rice	Wheat	Maize	Top 3	Rice	Wheat	Maize	Cassava	Sorghum	Millet	Top 6	Rice	Wheat	Maize	Top 3
Percentage energy of staple	42%	24%	5%	71%	15%	12%	21%	15%	8%	5%	76%	11%	16%	19%	46%
Staple as a % of top 3 staples (Asia, LAC) & top 6 (Africa)	59%	33%	7%	100%	19%	15%	28%	20%	11%	7%	100%	23%	35%	42%	100%
Iron (Fe)	35%	65%	9%	108%	12%	13%	39%	7%	15%	8%	95%	9%	16%	22%	47%
Zinc (Zn)	34%	39%	5%	78%	13%	9%	24%	7%	6%	5%	64%	9%	10%	16%	35%
Thiamine (B1)	49%	35%	8%	92%	18%	11%	39%	17%	10%	10%	106%	12%	13%	20%	46%
Riboflavin (B2)	10%	18%	3%	31%	4%	4%	16%	7%	2%	6%	38%	3%	4%	7%	14%
Niacin (B3)	78%	54%	7%	139%	27%	9%	33%	15%	13%	11%	108%	18%	9%	14%	42%
Pantothenic acid (B5)	59%	32%	3%	93%	21%	7%	11%	5%	4%	6%	55%	15%	8%	6%	29%
Vitamin B6	54%	16%	10%	80%	19%	3%	48%	13%	8%	8%	98%	13%	3%	30%	45%
Folate (B9)	8%	13%	2%	23%	3%	6%	9%	18%	4%	6%	46%	2%	9%	10%	21%
Calcium (Ca)	8%	5%	0%	14%	2%	2%	1%	4%	1%	0%	9%	2%	2%	12%	16%
Copper (Cu)	83%	92%	10%	186%	30%	22%	50%	31%	12%	32%	176%	20%	24%	28%	73%
Potassium (K)	11%	14%	2%	27%	4%	3%	10%	17%	4%	2%	40%	3%	3%	8%	13%
Magnesium (Mg)	48%	53%	10%	111%	20%	10%	51%	16%	17%	13%	127%	13%	9%	30%	52%
Manganese (Mn)	189%	198%	5%	393%	77%	34%	25%	38%	23%	24%	220%	50%	30%	16%	96%
Sodium (Na)	0%	0%	0%	1%	0%	0%	2%	1%	0%	0%	4%	0%	0%	0%	1%
Phosphorus (P)	59%	62%	8%	129%	21%	13%	33%	8%	14%	11%	100%	15%	15%	32%	62%
Selenium (Se)	104%	52%	10%	165%	38%	39%	43%	3%	15%	2%	140%	26%	55%	27%	108%
Vitamin A	0%	0%	0%	0%	0%	0%	1%	0%	0%	0%	2%	0%	0%	0%	0%
Vitamin C	0%	0%	0%	0%	0%	0%	0%	43%	0%	0%	43%	0%	0%	0%	0%
Vitamin D	0%	0%	0%	0%	0%	0%	0%	0%	0%	0%	0%	0%	0%	0%	0%
Vitamin E	4%	6%	1%	11%	2%	1%	5%	3%	2%	0%	13%	1%	1%	1%	3%
Vitamin K	1%	4%	0%	5%	1%	1%	1%	7%	0%	0%	9%	0%	0%	0%	1%

Abbreviation: LAC, Latin American countries.

The contributions of 9 staple crops (maize, wheat, rice, cassava, sorghum, millet, beans, potatoes, sweet potatoes) to total apparent per capita dietary energy intake were computed by country. Multiple typologies of the greatest contributions of staples to dietary energy were constructed by region. Staples identified in the typologies across each region are shown in the table. From among the countries included in each region, the population-weighted average percent contributions of each staple to total population-weighted daily dietary energy and micronutrient requirements based on the Harmonized Average Requirements [40] are shown.

TABLE 3, TABLE 4 show that for Africa and Asia, the primary food staples provide both a very significant proportion of the total intake (>50% in most instances) of iron, zinc, thiamine (vitamin B1), niacin (vitamin B3), pantothenic acid (vitamin B5), vitamin B6, copper, magnesium, manganese, phosphorus, and selenium. Moreover, these absolute levels contribute substantially to vitamin and mineral requirements. Food staples also contribute important amounts of riboflavin (vitamin B2), folate, calcium, and potassium to diets.

Percentage contributions of these nutrients are lower (but still quite significant) for Latin America where incomes are higher and diets are more diversified. By contrast, in all 3 regions, staple foods do not contribute significantly to the intakes of vitamins A, C, D, E, and K, with the exception of vitamin C in cassava.

In summary, these data show that food staples provide a very significant base pool of a range of minerals and vitamins in diets in LMICs, especially for low-income groups. Therefore, it is critical to address influencing factors and interventions that either improve or harm nutrient density in food staples.

**FIGURE 2:**
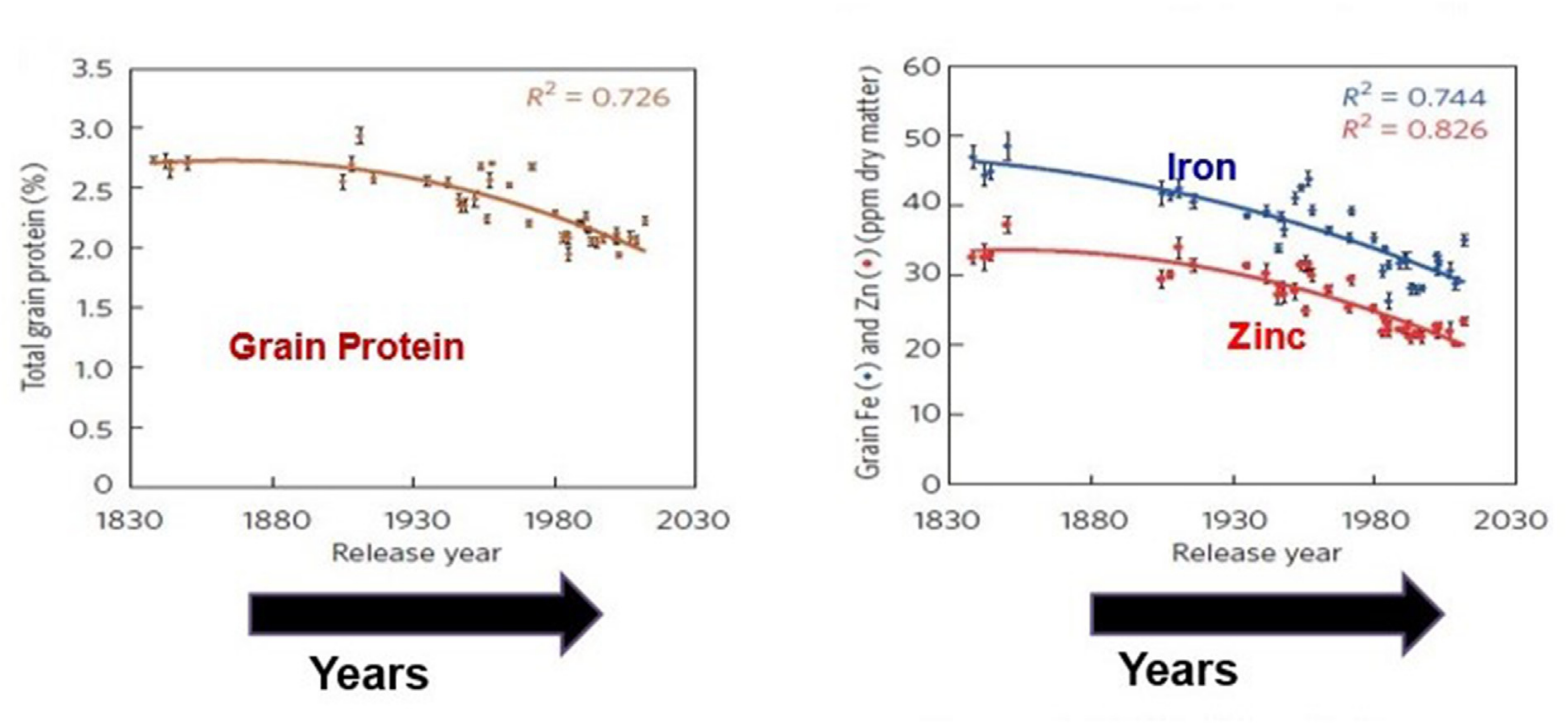
Trend in lower grain density. Historical changes in wheat grain protein and grain zinc and iron concentrations (with increasing grain yield). (Reproduced with permission from reference [41].)

For example, it is concerning that historical plant breeding efforts for food staples have not taken account of mineral and vitamin density (Figure 2) [41]. As yields have risen, nutrient densities have likely fallen. In addition, weakened resilience in food plants resulting from higher concentrations of carbon dioxide in the atmosphere and higher temperatures associated with global warming may affect the nutrient density of staple crops [42]. It is projected that the content of iron, zinc, and other nutrients will be depleted by 3% to 17% in most plants. Improvements in crop micronutrient density can therefore help offset climate-induced declines in productivity and nutrition [[42], [43], [44]].

## Nutrition Efficacy and Effectiveness

Providing rigorous evidence to convince the nutrition community (see key question 2 in the introduction) that biofortification has the potential for significant public health impact has been a 15- to 20-years process. First, it was necessary to determine the feasibility of breeding for particular nutrient levels (breeding targets) in specific crops. The second step was developing the nutrient-dense crops for testing. Finally, human nutrition trials were conducted.

### Setting mineral and vitamin density targets for plant breeders

Nutrient density targets were set for iron, zinc, and vitamin A such that biofortified crops could provide an extra 30% to 40% of the estimated average requirement (EAR) for nonpregnant, nonlactating women and preschool children when substituted 1-for-1 for their nonbiofortified counterparts in diets where these crops were consumed as a major staple food. (EAR targets for zinc were adjusted to 25% when the physiological requirements for zinc were revised substantially upward in 2014 [45], a moving target to which plant breeders have had to adapt. Fifty percent was used as an upper range, especially for provitamin A crops where breeding target levels were achieved most quickly [46]). These minimum targets provided a basis for exploring whether breeding for nutrient density could have a measurable impact on human nutritional status—a key metric of success.

The nutrient targets were set considering the baseline micronutrient content and natural variability of the nutrient concentration in the staple crop, the physiological nutrient requirement, per capita consumption of the staple food, the bioavailability of the nutrient, and nutrient losses during storage, processing, and cooking [1] (see Table 5).

**TABLE 5:** Target increments for plant breeding.

Crop	Target increment	% of target increment in released varieties to date	Proportion of EAR for nonpregnant, nonlactating women provided by crop (per capita consumption)		Proportion of EAR for children aged 1–6 years provided by crop (per capita consumption)	
			Before biofortification	After biofortification	Before biofortification	After biofortification
Beans	+44 ppm iron	50%–114%	50%	90%	40%	75%
			(200 g/d)		(65 g/d)	
Cassava	+15 ppm provitamin A	50%–112%	0%	>100%	0%	95%
			(500 g/d)		(250 g/d)	
Maize	+15 ppm provitamin A	50%–100%	0%	55%	0%	60%
	+12 ppm zinc	50%–92%	45%	70%	55%	80%
			(290 g/d)		(170 g/d)	
Pearl millet	+30 ppm iron	50%–103%	50%	85%	45%	75%
			(220 g/d)		(70 g/d)	
Sweet potato	+32 ppm pro vitamin A	50% to >100%	0%	>100%	0%	>100%
			(400 g/d)		(200 g/d)	
Rice	+12 ppm zinc	50%–92%	40%^1^	70%^1^	40%^1^	70%^1^
			(400 g/d)		(150 g/d)	
Wheat	+12 ppm zinc	50% to >100%	49%	73%	24%	35%
			(300 g/d)		(70 g/d)	

Abbreviation: EAR, estimated average requirement.

1 Contributions to zinc requirements are based on the European Food Safety Authority (EFSA) dietary reference values for zinc.

To provide empirical estimates for these calculations, HarvestPlus funded a series of studies for each crop, undertaken by collaborating institutions: *1*) household surveys in selected rural settings in target countries to estimate baseline dietary intakes and storage, processing, and cooking practices for the primary staple foods; *2*) nutrient retention studies under typical storage, processing, and cooking conditions; *3*) nutrient bioavailability and absorption studies; and *4*) randomized controlled efficacy trials and effectiveness studies.

### Retention, bioavailability and absorption, and efficacy trials

The prerequisite retention, absorption, and bioavailability studies with iron-, provitamin A-, and zinc-biofortified crops demonstrated that the breeding targets were achievable. Nutrients were absorbed in sufficiently greater amounts from biofortified crops than from their nonbiofortified counterparts [47]. Despite losses during storage, processing, and cooking, nutrient retention studies showed that biofortified crops could contribute significantly to women and children’s physiological needs for iron, zinc, and vitamin A [[48], [49], [50], [51]].

Subsequent efficacy and effectiveness studies sought to evaluate not only nutritional outcomes but also functional health outcomes. The trials show that increases in intake of iron, zinc, and provitamin A from eating biofortified foods results in significant improvements in nutrition and health for women, adolescents, and children; the results of these randomized controlled trials are comprehensively covered in [1].

Some examples of demonstrated nutrition and functional health impacts from consuming biofortified crops include:

• Iron beans, iron pearl millet, and iron rice improved iron status [[52], [53], [54]].
• Iron beans and iron pearl millet improved cognitive function and work performance in women and children, respectively [[55], [56], [57], [58], [59]].
• Provitamin A maize and provitamin A cassava improved vitamin A status [60,61], as did provitamin A sweet potato [62,63], with impacts sustained for years [29,33,64,65].
• Zinc wheat or zinc rice have not improved zinc status as measured by serum zinc; a reliable alternative biomarker to serum zinc has not yet been established [66,67].
• Zinc wheat and provitamin A sweet potato reduced levels of morbidity [5,68].
• Zinc rice modestly improved children’s growth [67].

From a historical perspective, these studies have transformed our understanding of the contributions that minerals and vitamins in food staples can make to micronutrient status. For iron in particular, findings have countered concerns from single-meal studies that bioavailability would be as low as 1% to 2% due to the high phytate content of staple foods [69]. Studies demonstrated that the bioavailability of iron in iron-biofortified crops ranged from 5.0% to 9.2% [70,71]. The impact of iron absorption inhibitors is much less pronounced in a mixed diet, the body adapts over time to absorbing the iron it requires from the diet, and percent bioavailability is dependent upon individual iron status—the lower the baseline iron status, the more iron absorbed [72].

Moreover, in biofortified staple foods, provitamin A has converted much more efficiently to retinol equivalents than originally anticipated. The provitamin A to vitamin A equivalency ratio is 4:1 for provitamin A cassava and 3:1–7:1 for provitamin A maize (compared to a range of 10–80:1 for vegetables [73,74]).

Plasma zinc concentration (PZC) is the biomarker most used to measure population zinc status, yet PZC results are easily confounded. For individuals, a reliable clinical biomarker remains elusive for anything other than severe deficiency. Several novel biomarkers are showing promise in clinical studies [[75], [76], [77], [78]]; their use in future studies may help reveal subclinical zinc deficiency that could be controlled with timely nutrition interventions to mitigate the global burden of zinc deficiency [79].

Given this body of evidence, global public health and nutrition bodies endorse biofortification as an efficacious intervention to improve nutrition based on the peer-reviewed evidence available. The Food and Agricultural Organization of the United Nations considers biofortification a complementary intervention that can improve micronutrient intake and contribute to healthy diets; the WHO recognizes biofortification as a sustainable strategy to be included in country’s food and nutrition programs; the 2021 *State of Food Security and Nutrition in the World* report supports biofortification to improve nutrient availability; and the Micronutrient Forum calls for the urgent scale-up of biofortification, among several other evidence-based interventions [[80], [81], [82], [83]].

## Crop Development, Variety Release, and Uptake

This section addresses the third and fourth key questions raised in the introduction: could plant breeding be successful in combining nutrient density with high yields? Would farmers adopt the new biofortified varieties, replacing old varieties, and would consumers buy and consume them in significant quantities?

### Crop development and variety release

In the 1990s, there was considerable skepticism among plant scientists that high nutrient density could be combined with high yields. There was a presumption that there would be a “win-lose” trade-off such that high nutrient density would be associated with low yield. This in fact had been the experience with high quality protein maize (protein being a macronutrient), CGIAR’s first attempt at biofortification [84]. If this were the case, farmers would not adopt low-yielding biofortified crops for their high nutrient density. The strategy would fail.

However, one emerging line of research has shown that increasing seed zinc density gave seedlings greater vigor and resulted in better stand establishment, improved ground cover, reduced evaporative moisture losses, and hence, higher yields. Both healthy plants and healthy humans depend on a range of trace minerals [[85], [86], [87]]. This potential “win-win” was the justification upon which the pipeline of higher yielding, nutrient-dense biofortified crops was developed.

The process and outcomes of 2 decades of crop development research on biofortification is documented in [1]. Crop accession core collections in Center germplasm banks along with breeding program materials were screened for variation in nutrient density. Based on findings and given the target of adding 30% to 40% of the EAR, single nutrients were identified for a number of crops that gave the highest probability of success (Table 5). In this process, crop breeders transfer otherwise untapped trait variations from underutilized plant varieties and landraces, increasing genetic agrobiodiversity.

**FIGURE 3:**
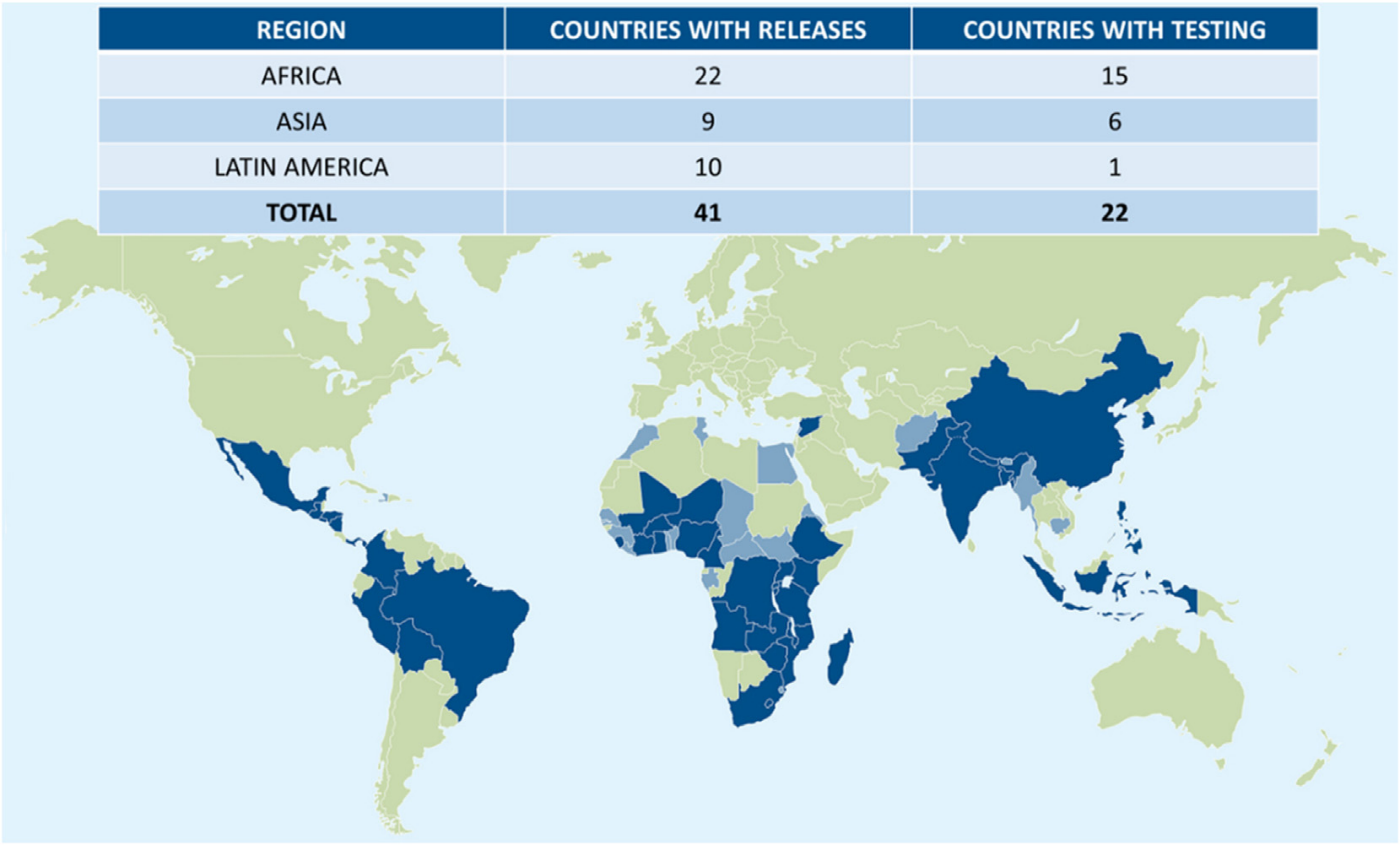
Global reach of released varieties and varieties in testing for release. Dark blue countries: Countries where biofortified varieties are released. Light blue countries: Countries where biofortified varieties are in testing.

By 2024, nearly 450 biofortified varieties of 12 crops had been released in 41 countries and were in testing for release in an additional 22 countries (Figure 3, Table 6). Biofortified crops are approved for release by national agricultural research systems based on their proven ability to meet agronomic standards. Nonetheless, the misconception is still held that biofortification imparts a yield penalty [88].

**TABLE 6:** Number of countries with biofortified crop releases and number of releases, by crop, as of December 2023^1^.

Crop	Africa		Asia		Latin America		Total	
	Number of countries	Number of varieties released	Number of countries	Number of varieties released	Number of countries	Number of varieties released	Number of countries	Number of varieties released
Beans	8	54	0	0	8	25	16	79
Cassava	5	25	0	0	1	3	6	28
Maize (vitamin A)	12	77	0	0	1	1	13	78
Rice	0	0	3	14	4	4	7	18
Wheat	0	0	4	29	3	3	7	32
Pearl millet	2	2	1	12	0	0	3	14
Sweet potato	17	112	5	18	6	16	28	146
Others^2^	2	14	6	20	6	14	14	48
Total		284		93		66		443

1 Statistics are for Consultative Group on International Agricultural Research Biofortification Strategy which includes International Potato Center activities for sweet potato.

2 Maize (Zn), cowpea, sorghum, banana/plantain, lentil.

Multiple biofortified varieties of specific crops have been released in single countries; some countries have released biofortified varieties across multiple crops (e.g., iron pearl millet, zinc wheat, zinc rice, and vitamin A sweet potato in India). Table 6 tallies country-crop combinations by crop by region.

### Uptake of biofortified crops by farmers and consumers

Iron beans, provitamin A cassava, provitamin A maize, and provitamin A sweet potato have had primary impacts in Africa while zinc rice, zinc wheat and iron pearl millet have had primary impacts in Asia. Starting in 2010 and with varied starting years, HarvestPlus has secured funding for and undertaken focused scale up activities in 13 countries in Africa and Asia:

• Africa (beans, cassava, maize, pearl millet): DR Congo, Kenya, Malawi, Nigeria, Rwanda, Tanzania, Uganda, Zambia, Zimbabwe
• Asia (rice, wheat, pearl millet): Bangladesh, India, Indonesia, Pakistan

HarvestPlus has monitored and modeled the uptake of biofortified crops in these 13 countries using the Global Households Reached Projections Model. This model takes into consideration the number of farm households buying or receiving program seed in a particular year, farmer to farmer diffusion, and disadoption/attrition, among other factors [89]. Model projections of numbers of on-farm and off-farm consumers may be found on the HarvestPlus website [90].

The best documented impact is for iron beans in Rwanda where HarvestPlus undertook a nationally representative survey of bean-producing households. Yields of biofortified beans were estimated to be 23% higher for bush beans and 20% higher for climbing beans as compared with nonbiofortified bean varieties [91]. In Pakistan, a zinc wheat variety, Akbar 19, is fast becoming the most widely grown variety due to its superior yield and tolerance to heat stress [92]. (The rapid uptake of Akbar 19 is too recent to be posted in government statistics. If Akbar 19 is planted on 42% of wheat area, then given its higher-than-average yield, production could approach half of the wheat supply in Pakistan in 2024. This would imply well over 100 million Pakistani consumers of Akbar 19.) Additionally, recently released zinc wheat varieties are gaining momentum and diversify the biofortified varietal mosaic.

Iron and zinc are invisible micronutrients that do not affect taste. In Nigeria, between 1 and 2 million farm households for each crop have adopted production of provitamin A (yellow) cassava and (orange) maize [93]. Change in color has not been a barrier to adoption. There is extensive literature on willingness to pay for nutrient-dense crops under varied levels of information explaining the color change [94,95].

Details of strategies for scaling up specific types of biofortified crops (vegetatively propagated, self-pollinating, and hybrid seeds; invisible iron/zinc and yellow/orange vitamin A) are provided in [1], articles reviewed in Foley et al. [96], Birol and Bouis [97], and chapters in Bouis [98], and specifically for orange sweet potato in Low and Theile [99], Low et al. [100], and Lindqvist-Kreuze et al. [101].

HarvestPlus has invested in working closely with the agricultural research institutes in Brazil, China, and India to promote the development of domestically-funded biofortification programs in these 3 countries with very large and regionally influential agricultural research institutions [102,103]. Strategic partnerships for scaling were also forged, such as the delivery partnership between HarvestPlus and World Vision which started in 2014 and is ongoing. With technical assistance from HarvestPlus, World Vision—through its existing agricultural programs implemented with local nongovernmental organization partners—has taken the lead in scaling up the consumption of biofortified crops in >17 countries. From 2019 to 2022, HarvestPlus and the Global Alliance for Improved Nutrition collaborated on the Commercialisation for Biofortified Crops project, to scale up the number of consumers of nutrient-rich biofortified foods in Africa (Nigeria, Kenya, and Tanzania) and Asia (India, Bangladesh, and Pakistan) through commercial pathways.

In summary, the evidence of widespread release and adoption of biofortified varieties globally puts to rest the third and fourth seminal questions listed in the introduction as to the productivity of biofortified crops and the potential for acceptance by farmers and consumers. Key issues as to whether this momentum will be sustained and deepened are discussed in a later section.

## Influence on Policy Linking Agriculture and Nutrition

The nutrition community at the World Bank recognized that investment in nutrition was limited and embedded in health, but the impact of malnutrition was pervasive on economic outcomes. Their seminal work repositioned nutrition in development by revealing the costs of undernutrition in terms of gross domestic product and a range of other developmental outcomes [104]. The Copenhagen Consensus 11 project ranked biofortification fifth of the 15 highest-rated opportunities to address the 10 most pressing challenges based on returns on investment and third with respect to addressing the nutrition challenge [105].

In 2013, the World Bank followed up on its repositioning argument for nutrition with a call to invest in multisectoral approaches, recognizing that the solution should not be health-focused alone [106]. In this report, it highlighted 3 distinct ways by which agriculture could increase nutritious food production, the second being biofortification:

• Adding the production of specific nutrient-dense foods, such as fruits and vegetables, fish, and livestock;
• Increasing nutritional content of the food produced, e.g., through crop biofortification, mineral fertilization, and industrial food fortification;
• Improving the preservation of nutritious food for year-round access and eliminating seasonal food shortages.

There was a focus on increasing the capacity of national agricultural research institutions to promote the breeding for and dissemination of biofortified crop varieties, recognizing that staple foods are more resilient and increase year-round access to more nutrients [106]. This work drove a focus on nutrition by the agricultural sector, with the ultimate goal of delivering an affordable, accessible, year-round nutritious food supply.

Biofortification is a relatively easy fit for agriculturalists, who often have very limited nutrition skills. Many agricultural programs focus on staple crops because these are demanded by governments who recognize the risks of failing to deliver affordable, accessible staple foods to their citizenry. Designing nutrition-smart agricultural programs that focus on productivity enhancement and also nutrition, immediately suggest adoption of biofortification, and require no nutrition skills for the agricultural implementers. Growing biofortified staple crops will automatically increase micronutrient supply in the staple food system, and when nutrition training and behavior change are incorporated, that impact is magnified [107,108].

In 2014, at the Second Global Conference on Biofortification, and quoted in the Global Panel on Agriculture and Food Systems for Nutrition brief supporting biofortification in 2015, Akinwume Adesina, President of the African Development Bank and member of the Global Panel, said: “The challenge is no longer the science of biofortification—we know it works; our challenge as policy-makers is to scale up biofortified crops to reach millions of households through institutional, regulatory and financial policy” [109].

The African Union (AU) recognized the potential impact of biofortification across the continent to support their nutrition and agricultural strategies. The AU Heads of States adopted the Declaration on Scaling-up Food Fortification and Biofortification in Africa, which was closely aligned with the second point made by the World Bank above, at the 40th Ordinary Session of the Executive Council of the African Union, held on 2–3 February, 2022 in Addis Ababa (EX.CL/Dec.1143-1167(XL)). This resulted in integration of the African Biofortification Priority Index in the Comprehensive African Agricultural Development Programme Biennial Review process, which also reports to the Heads of State.

The other two pillars with respect to the biofortification declaration are advocacy and access to affordable, quality biofortified seeds. The AU has taken advocacy on board and included biofortification in its Africa Common Position Paper to the United Nations Forum on Food Systems [110], as did other countries, including Ethiopia [111]. The AU is partnering with CGIAR, notably SeedEqual, to support the intensification of the biofortified seed system.

Consistent with the AU declaration, numerous African countries have included biofortification in their agricultural and nutrition programs. For example, Nigeria instigated a directorate for Nutrition and Food Safety in their Agricultural Ministry, operationalizing it in 2023. It has committed to increasing adoption of both more biofortified crops and increased area under cultivation for existing adopted crops [112].

A number of countries are pursuing strategies to incorporate biofortified foods into school feeding programs, generally pursuing virtuous circles of local production and procurement (Kenya, Tanzania, Malawi, India, Nigeria, Honduras, Columbia, Zambia, and Zimbabwe). Most countries have industrial food fortification guidelines, most often led by the Ministry of Health, and revised every 5 years. Zimbabwe has led the way as the first country with its finalized, adopted revision in 2023, dedicating equal weight to both industrial fortification and biofortification. With regard to seed standards, national level examples include the minimum iron and zinc breeding targets set for pearl millet in India and iron bean seed and grain standards in Rwanda [113]. Publicly available specifications published by the British Standards Institution for iron-, zinc-, and vitamin-A enriched grains determine the amount of a micronutrient required within a crop for it to be designated biofortified or nutrient-enriched. These standards are applicable globally and include requirements for concentrations of nutrients, sampling, packaging, and labeling of biofortified products [[114], [115], [116]].

At the international level, several UN agencies have integrated biofortification in their recommendations and programs. The World Food Programme was a first mover that integrated biofortified seeds into their Purchase for Progress program as far back as 2011–2012. High iron beans grown in Rwanda were used to feed refugees in the Democratic Republic of Congo. Biofortification was included in UNICEF’s the State of the World’s Children 2019 Report [117] and in World Food Programme’s local and regional food procurement policy [118].

Several international financial institutions now have nutrition targets for their loans to the agriculture sector, and biofortification is considered as a cost-effective and readily implementable technology that requires minimum behavior change and infrastructure investment. Biofortification is included in the International Fund for Agricultural Development’s Nutrition Sensitive Value Chains guidelines [119] and in African Development Bank’s Multi-Sectoral Nutrition Action Plan [120]. World Bank documents describe means to deliver nutrition sensitive agriculture [121] indicating biofortification as a prime example of nutrition-smart agriculture investments [122]. An increasing number of World Bank loans include investments in the scale-up of biofortified crops (see examples in Malawi [123], Uganda [124], and the Democratic Republic of Congo [125]).

## Challenges Moving Forward

The term “newly emerging” technology is used in the title of this article for a good reason. Development, introduction, and adoption of new crop varieties—whether staple crops or nonstaple foods—have long lead times. For example, CGIAR Centers began initial operations in ∼1960 to raise staple crop yields; only by 1980 did the impacts of these activities begin to take effect. Sustained donor and policymaker interest over 4 decades is required to integrate biofortification into the core activities of agricultural research centers globally, for the international nutrition community and agricultural policymakers to value and advocate for biofortification, and for consumers to become aware of the nutritional value of enriched staple food crops. Significant hurdles overcome during the past 20 years and challenges moving forward over the next 2 decades are discussed here.

### Historical context: hurdles overcome

At the initial CGIAR meeting in 1994, as was mentioned in the introduction, it was agreed that breeding for higher mineral and vitamin densities in the edible portions of cereals and root crops had sufficient potential that it was worth pursuing. Yet to do so would require substantial funding and resolution of several scientific and implementation issues. Herein lay the funding challenge: agricultural donors were not willing to fund human nutrition objectives, and nutrition donors were not keen to invest in agriculture.

Seed funding provided by the Danish International Development Agency and the Asian Development Bank (USD $5–6 million in total) enabled 8 years of investigative research that kept the idea of biofortification alive. During this time, a second follow-up meeting was convened at the International Rice Research Institute in the Philippines attended by equal numbers of plant scientists and human nutritionists; the outcome was a special issue on improving human nutrition through agricultural research [126]. In a major breakthrough at the end of 2002, the Biofortification Challenge Program (BCP) was approved by CGIAR donors, with inaugural funding primarily from the World Bank. These Challenge Programs were an initial attempt at major CGIAR reforms.

In 2003, the Bill & Melinda Gates Foundation (BMGF, which to that time was investing in global health and not agriculture) approved a 4-year USD $24 million grant to the BCP, which shortly thereafter was renamed as “HarvestPlus.” With this funding, it was now possible to initiate a process to develop, test, and deliver biofortified crops to farmers and consumers. However, to achieve impact, funding would need to be sustained over a number of years. Ten years is a benchmark figure for building a plant breeding pipeline for a specific crop; this time is required to undertake the necessary steps from initial germplasm screening to approval by national varietal release committees. Only then can seed multiplication and outreach to farmers and consumers begin.

**FIGURE 4:**
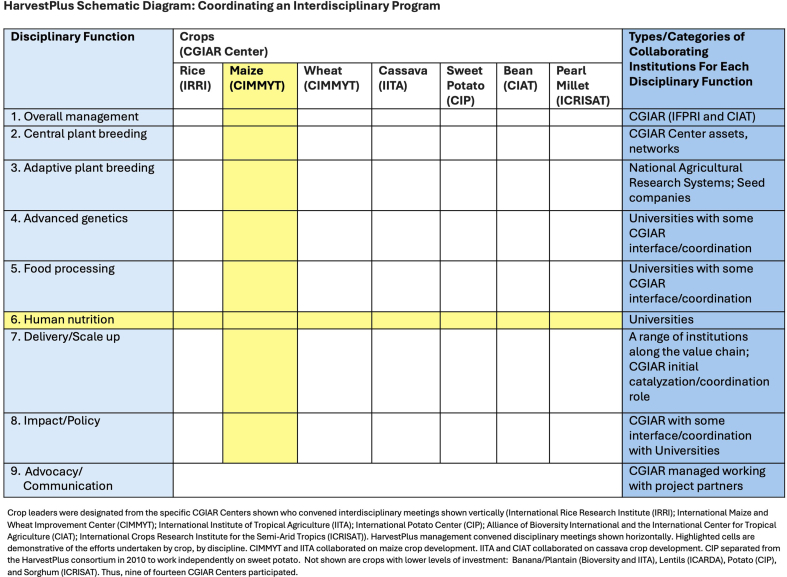
HarvestPlus schematic diagram: coordinating an interdisciplinary program.

From its inception, HarvestPlus organized its biofortification activities around the matrix shown in Figure 4. This structure allowed efficient coordination of activities and the free flow of information among collaborators. With oversight through the HarvestPlus governance structure, “core” funding was allocated by HarvestPlus managers to collaborating institutions for activities to be undertaken in each cell. Individual crop meetings were held that brought together scientists and implementing agencies across multiple disciplines. Intradisciplinary meetings were also held across crops to discuss biofortification successes, failures, and future plans.

Over the first 20 years of HarvestPlus, ∼60% of total funding of USD $500 million came from the BMGF and the UK aid agency (then referred to as the Department of International Development) in equal proportions—most of it invaluable core funding which could be spread across the cells in Figure 4 as determined by HarvestPlus management. Other relatively large donors have been CGIAR central funding mechanisms, Canada, the United States of America, World Bank, and the MacArthur Foundation, contributing a combined 30% of total funding.

### Criticisms of biofortification

As biofortification has emerged as a widely-accepted and expanding strategy, criticisms have appeared [88,127]. Van Ginkel and Cherfas [88] make the following primary points:

1. There is a yield penalty, i.e., there is a tradeoff between mineral/vitamin density and food staple productivity so that farmers will not adopt biofortified varieties.
2. There is scant nutritional evidence that biofortified crops are efficacious and effective.
3. Resources and attention that have been devoted to biofortification have held back progress on strategies to expand vegetable and fruit production and their intake.

The large body of literature on nutritional bioavailability, efficacy, and effectiveness and on variety development, release, and uptake by millions of farmers globally refute the first 2 points.

Regarding the third point made by Van Ginkel and Cherfas [88], resource allocation to biofortification must be compared to the magnitude of the task at hand and the measurable benefits (cost-benefit analysis). There is no comprehensive accounting of global investments in biofortification. HarvestPlus spent ∼USD $500 million over its first 20 years, or ∼$25 million per year to improve the nutritional quality of several major food staples globally in LMICs, to undertake foundational nutrition research related to biofortification, and to promote the uptake of biofortified foods by farmers and consumers. When compared to other long-standing and efficacious micronutrient interventions, it is clear that the investment in biofortification has been relatively small. For example, 500 million vitamin A capsules are distributed annually to preschool children at a cost of USD $1 to $2 per capsule or nearly USD **$1 billion per year** in current dollars. (See Supplementary Material 2 for an explanation of these cost figures.) Benefit-cost studies indicate that several multiples of USD $25 million per year are paid for by the benefits of biofortification.

Maliézieux et al. [127] make the primary argument that in the long analysis, a more diversified agricultural production system solves nutritional and sustainability goals that staple food production alone cannot address. The biofortification strategy does not fundamentally misalign with this view. Biofortification very simply contributes to reducing some nutrient deficiencies cost-effectively and can be implemented alongside other complementary nutrition interventions. Food staple production is necessary to stave off hunger and contributes significantly to mineral and vitamin intakes. Both Van Ginkel and Cherfas [88] and Malézieux et al. [127] would be well served to undertake economic analysis to understand the enormous magnitude of the investment required under various sub-strategies for which they advocate to achieve diversified production systems and diets in the LMICs.

Biofortification should be viewed as one component of a unified package of interventions that together seek an end to malnutrition. Various groups are attracted to working on specific types of interventions which in the end complement one another. Each group must justify its own impact and return on investment to sustain both donor and policymaker interest. In the case of rice and wheat, for example, the responsibility for developing more productive varieties tends to rest on the public sector until commercially viable hybrid varieties can be developed. In the sphere of hybrid vegetables, the expanding activities of the East-West Seed company with a focus on smallholder farmers have been rightly recognized by the World Food Prize.

### Moving along the current trajectory

The trajectory from the concept of biofortified crops to their consumption across Africa, Asia, and Latin America over 20 years was realized under the management structure outlined in Figure 4. Due to declining funding for HarvestPlus and ongoing reorganizations of CGIAR activities, as of 2021, this management structure is no longer in place. The UK government has significantly reduced its foreign aid budget. There is an element of donor fatigue and agriculture managers at BMGF give high priority to productivity and climate smart crop traits, as do many CGIAR managers. Under the currently operating CGIAR reform, no central funding has been allocated to HarvestPlus. A follow-up reform that is currently being discussed will be implemented in 2025.

To continue biofortification, it is now the responsibility of collaborating institutions in each cell in Figure 4 to secure funding and independently synchronize their activities with others. HarvestPlus funding now is largely designated for scale-up activities in 8 African and 4 Asian countries with 9 donors underwriting these activities for contributor-specific countries of interest (short-term returns on investment). This altered structural environment renders sustained long-term investment and coordination very difficult. Leadership is virtually impossible. It may well be that the final question raised in the introduction as to sustained donor and policymaker interest is answered in the negative.

#### Sustaining the breeding pipeline

In 2003, agricultural donors and CGIAR Center managers took a conservative approach to biofortification. At each Center, separate breeding pipelines were set up for biofortified crops, which were not initially included in the main Center breeding programs, an approach referred to as targeted breeding. The view was that once it was proven that biofortification can have significant impacts, mineral and vitamin traits would be “mainstreamed” into Center breeding programs.

Mainstreaming has now begun for zinc for rice and wheat, but may take a decade and will require investment, perseverance, and priority. In retrospect, integrating biofortification into core breeding programs has taken too long. Moreover, targeted breeding needs to continue as a bridge until mainstreaming is fully implemented.

Progress using conventional breeding is a stepwise process. Certain gains in increasing nutrient density then are combined with high yields and other traits, giving a higher “platform” from which to realize further gains in nutrient density and again combining this advance with even higher yields—and the process continues.

Provitamin A maize provides an instructive example of how nutrient densities can increase over time, building on the stepwise nature of plant breeding mentioned above and as knowledge increases and experience is gained, and of how sustained, long-term support for agricultural research is crucial.

Originally, a target of 15 μg/g provitamin A carotenoids was set for maize at harvest to provide an estimated additional 40% of the EAR after considering losses in provitamin A in storage, processing, and cooking. Slow breeding advancement in the first years of HarvestPlus led to some consideration of dropping orange maize from the program; target levels appeared unattainable. However, progress accelerated by using tropical parents with higher levels of provitamin A, and research on genes controlling the carotenoid pathways allowed effective application of marker-assisted selection [128].

Table 5 shows that orange maize varieties released to date have achieved 50% to 100% of the target value of 15 μg/g. Dietary information was collected from a sample of 30 farm households adopting orange maize in Zimbabwe. Vitamin A adequacy improved but fell significantly short of attaining 100% of requirements [129].

More recently, as more has been learned about the interaction of favorable alleles along the carotenoid pathway, development of inbred maize lines with provitamin A carotenoid densities above 25 μg/g are common, with a maximum achieved of 50 μg/g. Moreover, densities of lutein and zeaxanthin (non-provitamin A carotenoids that contribute to vision and brain function) have been increased simultaneously [130]. Despite this impressive breeding progress, in this example the effort to reach farmers and consumers must stop due to lack of funding/priority to complete varietal development for release. This particular technology likely will remain on the shelf for some time.

Nevertheless, breeding pipelines no longer depend entirely on CGIAR centers. For example, the Indian national agricultural research system has given significant priority to biofortification [103] as have other national agricultural systems. Within India, during 2021–2022, biofortified varieties covered over 5.5 million hectares (ha), with a predominant focus on crops such as wheat (4.5 million ha), pearl millet (0.5 million ha), mustard (0.5 million ha), rice (0.1 million ha), and lentil (0.05 million ha) [131]. It may turn out that the required interdisciplinary activities can be funded and organized at the national level in some countries.

#### The role of consumer demand

Ultimately, if consumers demand biofortified varieties, food systems will provide them. White is the current norm for the color of food staples in LMICs, such as for cassava, maize, and rice. For provitamin A-enriched crops, as long as there is price equality between white and orange/yellow varieties, the value proposition to consumers is very strong. If governments and other institutions invest in and strongly back the message that yellow/orange is healthier, then it is more likely that orange/yellow can become the new norm. As already discussed previously, any biofortified crop must also be competitive yield- and taste-wise to compete with existing varieties [94,95].

Iron and zinc, which are tasteless and invisible at the levels added to food staples through plant breeding, are a different case. The most efficient path is to seek that as many staple varieties as possible are biofortified with iron and zinc, just as universal fortification of a commercial food is the least expensive strategy. The Indian Council of Agricultural Research has prioritized nutrition in their breeding program, setting out minimum levels for iron and zinc in all national pearl millet varieties [103,132].

#### Convincing agricultural policymakers to give high priority to human nutrition

A plaque in the lobby of FAO headquarters reads: “In this building, 16^th^ of October 1945, representatives of 44 nations met and established the FOOD AND AGRICULTURAL ORGANIZATION, first of the new United Nations Agencies. Thus, for the first time, nations organized to raise levels of nutrition and to improve production and distribution of food and agricultural products.”

The objective of improving human nutrition is mentioned first and agricultural supply second. This is no longer the norm for agricultural policymakers who give priority to agricultural productivity, income generation, and poverty reduction. Indeed, higher household income is the most potent driver of better, sustained dietary quality [37]. However, we produce food to be healthy, and policymakers should not turn a blind eye to cost-effective interventions such as biofortification, which can improve the nutrient quality of the food supply.

Agricultural policymakers are often unaware of the public health significance of mineral and vitamin deficiencies, driven by poor quality diets. The consequence is that agricultural policy does not take account of nutrition impacts, or this is given low priority. Raising awareness is key. Having scalable varieties and seed supplies readily available gives the opportunity to act relatively quickly once policymakers are convinced.

#### Long gestation periods and focus on specific foods

One important lesson that biofortification teaches is that policymakers and donors need to acknowledge at the outset that any specific agricultural strategy to improve nutrition will take decades to implement cost-effectively at scale across a number of countries—a process that involves raising the funding, doing the research, building consensus, changing policies, securing uptake by farmers and acceptance by consumers, and so forth.

Food systems are complex. How does one choose where and how to intervene, especially when resources are constrained? “Improve Food Systems” is too broad and general a message. Rather the focus should be on individual key nutritious foods (which will vary by country), and then to eliminate the specific bottlenecks which need to be overcome across the value chain for that food to be scaled up or mainstreamed into the food system—whether it be a biofortified food staple or a nonstaple food. For example, dietary quality also may be improved by increasing the national productivity of egg and milk production and of particular vegetables and pulses in specific countries, thereby lowering prices and increasing consumption of these foods [133,134].

### Shifting to a higher trajectory to multiply the impacts of biofortification

Interestingly, no mention is made in Van Ginkel and Cherfas [88] of the 2 primary drawbacks to biofortification, which are the long gestation periods of agricultural interventions to sustainable impact and, thus far, the addition of single nutrients to diets. Both shortcomings may be mitigated through the application of genetic engineering (GE).

The general concept of biofortification for application in LMICs is to piggyback on the latest high-yielding, climate smart varieties coming out of agricultural research systems and ultimately **to bundle together** several nutrition-improving traits in these varieties. This may be contrasted with giving consumers a choice in markets, for example, to buy a low-glycemic-index rice, for example **OR** a high provitamin A rice. Rather, the objective would be to breed low-glycemic-index rice varieties that are also relatively dense in provitamin A **and** iron **and** zinc **and so forth.** HarvestPlus started with provitamin A, iron, and zinc because there was consensus that these 3 were (and still remain) major public health problems. Using conventional plant breeding, unfortunately, it was not practical to breed for these three nutrients simultaneously. Plant breeders using conventional techniques prefer to approach each nutrient sequentially.

#### Breeding objectives: selection of additional nutritional traits

Optimizing the nutritional and health impacts of food staples holds a myriad of opportunities. As discussed previously, food staples provide a significant portion of a range of minerals, vitamins, and amino acids in diets. Their densities also may be increased as already demonstrated for provitamin A, iron, and zinc.

Certain compounds such as ascorbic acid, resistant starch, and some amino acids, if levels are increased, may improve the bioavailability of a range of trace minerals [135]. Other compounds such as phytic acid and some polyphenols inhibit trace mineral bioavailability; their density levels may be decreased [136]. (Some polyphenols may be promoters of bioavailability. Hart et. al. [137] reported the polyphenol kaempferol-3-glucoside is an iron promoter in beans.) In all cases, one must take account of possible negative linkages with plant yield and other beneficial roles in human nutrition apart from mineral and vitamin requirements and desirable phytochemical contents.

Resistant starches in rice are associated with a lower glycemic index that results from delayed/more digestion of resistant starch in the intestine, lower post prandial blood sugar spikes, and better gut health [138,139]. Plant breeding can also be a possible strategy for lowering aflatoxin levels in some food staples that have harmful health effects [140,141].

Advanced crop development techniques like gene editing and gene stacking allow for the bundling of several of these traits in single varieties at much more rapid speeds than possible by conventional breeding. It is evident that the choice of tools to achieve such bundling of quality traits in any crop should be rational, i.e., based on proven effectiveness to reach the required levels of micronutrients and other compounds and the speed at which the combination of traits can be obtained [142]. The regulatory environment in countries targeted for deployment, intellectual property rights, and access and affordability for poor farming households as well as related to the final product should also be considered.

#### Use of GE in development of biofortified crops

GE, also known as genetic modification, is a technology that allows introduction of adjustments as well as additions to the genetic code of an organism. Using this set of techniques, beneficial traits such as enhanced crop disease resistance or enhanced micronutrient content can be implemented. GE can be performed from the smallest scale—modifying one letter of the genetic code—to a larger scale, where a whole set of genes is introduced.

Provitamin A Golden Rice provides an instructive example. No rice variety contains provitamin A in the milled endosperm that is eaten. Thus, GE was the only option for developing provitamin A rice. Two biosynthetic genes, *PSY* and *CRTI*, were introduced into rice to create Golden Rice. The *PSY* gene originates from maize while *CRTI* originates from a common soil bacterium [143].

Unlike provitamin A, milled rice contains some iron and zinc. Research revealed that the densities of iron and zinc could be increased much more using GE than using conventional breeding techniques. Expressing dicot ferritins and the rice nicotianamine synthase 2 (*OsNAS2*) gene has resulted in relatively high levels of iron and zinc being transported to the endosperm from plant tissues [144,145].

Both the provitamin A and iron-zinc events have been combined separately in high-yielding varieties for field testing, and data have been generated for deregulation. The provitamin A event was deregulated in the Philippines in 2021 and is currently being commercialized. (On 17 April, 2024, the Philippine Court of Appeals issued a ruling—in a suit brought by Greenpeace and others—that not only should the 2-year-long commercialization of Golden Rice and Bt eggplant be stopped, but also that imports of genetically modified maize and soybeans be banned [146]. The case is under appeal by the Department of Agriculture, which has stated it will ignore the ban on imports. President Marcos has asked his Solicitor General to have the CA decision reversed [147]. A simplistic interpretation of the precautionary principle was invoked by the Court of Appeals [148]. During the court hearings, no specific evidence was provided that Golden Rice and Bt eggplant are harmful, only arguments that genetically modified organisms in general **might be** harmful.) The dossier for the iron-zinc event will be submitted soon for deregulation. The 2 events may be combined using conventional breeding techniques, giving a 3-in-1 rice with relatively high densities of all 3 nutrients. Because of the high yield and use of the same production inputs as for nonbiofortified rice, it can reasonably be expected that as the market grows for this 3-in-1 rice, it will sell in the same price range as nonbiofortified rice.

Table 7 [38,137,144,145,149,150] shows the estimated increases in densities (as consumed) and the additional amounts of vitamin A, zinc, and iron added to diets for an average consumption level of 300 g of milled rice per day for consumers in the Philippines. Table 7 also shows the average intake of vitamin A, zinc, and iron for the entire population and for the poorest income quintile. For any given day that the 3-in-1 biofortified rice is substituted 1-for-1 for nonbiofortified rice, vitamin A intake for the lowest income quintile is nearly doubled. For the total population, zinc intake is more than doubled and iron intake is increased by 25%.

**TABLE 7:** Increased intake per capita per day of provitamin A, iron, and zinc in milled rice in the Philippines.

Nutrient	Increment density^3^ (1)	Milled rice intake per day^4^ (2)	Increased intake per day^5^ (3)	Base intake average total population^4^ (4)	Base intake poorest quintile^4^ (5)
Provitamin A^1^	+1	300 g	+300 RAE	518 RAE	334 RAE
Zinc^2^	+31.6	300 g	+9.5 mg	9.0 mg	8.2 mg
Iron^2^	+7.8	300 g	+2.3 mg	9.7 mg	8.4 mg

Abbreviation: RAE, retinol activity equivalent.

1 Provitamin A = RAE per gram of milled rice before cooking (density); RAE/day (intake).

2 Iron and zinc = mg/kg (density); mg/day (intake).

3 Column 1: Vitamin A – density measured after milling and 2 mo of storage [149]; nonbiofortified white rice has zero vitamin A. Zinc and Iron – difference in density after milling between biofortified and nonbiofortified rices [144,145].

4 Columns 2, 4, and 5: Average per capita milled rice and nutrient intake; household-level 24-hour recall of food intakes [38].

5 Column 3: Result of multiplying columns 2 and 3; the assumption is a 1-for-1 substitution of biofortified for nonbiofortified rice [137,145,150].

**FIGURE 5:**
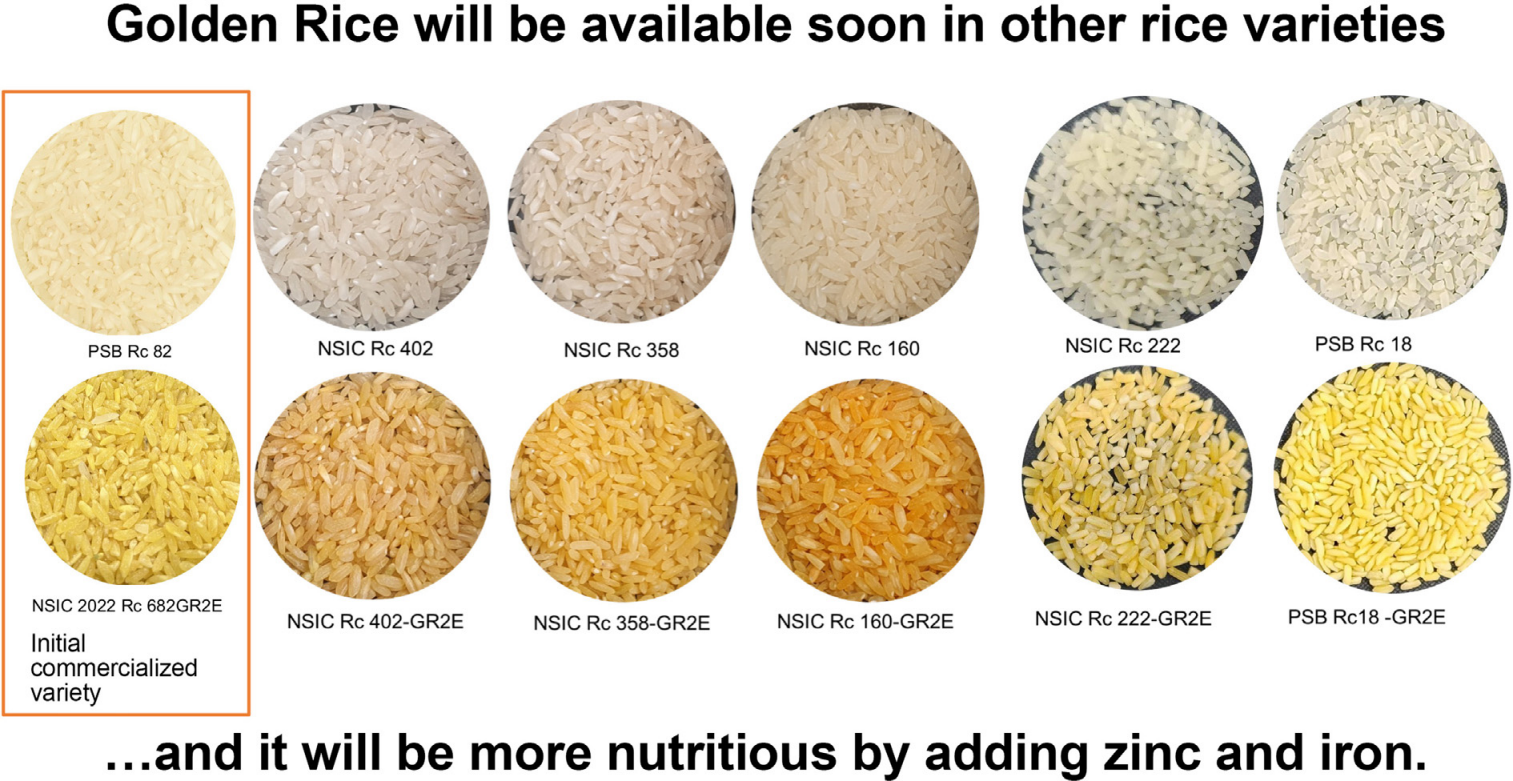
Multiple Philippine rice varieties with provitamin A will be available soon.

Now that the Golden Rice event is deregulated in the Philippines, conventional breeding is being used to cross the provitamin A genes into several popular, high-yielding Philippine rice varieties as shown in Figure 5. The first Golden Rice variety with a background of PSB Rc 82 was registered in 2022 with the National Seed Industry Council as NSIC 2022 Rc 682GR2E (with a local name Malusog 1, which means healthy). Additional stand-alone β-carotene Golden Rice will be registered in the following backgrounds: Rc 160-GR, Rc 222-GR, Rc 402-GR, Rc 358-GR, and Rc 18-GR. These are currently undergoing extensive multilocation field trials in selected PhilRice stations to generate agronomic and grain quality data, a prerequisite for the varietal registration. However, once the high-iron and high-zinc rice is deregulated, the plan is to stack it with Golden Rice to develop and register 3-in-1 rice varieties [149].

Note in Figure 5 that some of the varieties have a deeper orange color than others. As learned from the research experience with provitamin A maize, this may be due to a more favorable set of alleles along the carotenoid pathway in some varieties than others, giving a higher density and deeper color [150,151]. If the same is true in rice, then the provitamin A density shown in Table 7 can be increased further across several varieties just as has been accomplished with provitamin A maize.

This Golden Rice example demonstrates several advantages of the use of GE:

• Adding a nutrient to a food staple where none existed previously (provitamin A).
• Increasing the density of a nutrient in a food staple far more than has been possible with conventional breeding (iron and zinc).
• Stacking multiple nutrients in a single food staple variety.

Several more examples are discussed in more detail in [1]. Table 8 shows the comparative advantages of use of GE and conventional breeding. The combined enhancement of provitamin A, iron, zinc, and folates is a major target toward global improvement of hidden hunger in LMICs, given that these micronutrients are in the top 5 that must be derived from the diet [152]. Since GE has proven to be a robust approach to enhance and stabilize folates in rice as well as in potato [153,154], the strategy can be further combined with the genes successfully used to create Golden Rice [143].

**TABLE 8:** Comparison between breeding and genetic engineering (GE) to be used for micronutrient biofortification of crops^1^.

	Issue	GE	Breeding
Comparative advantages of GE	Tissue-specificity	Ability to control tissue-specificity	No adequate control on tissue-specificity
	Source of genetic material	Introduction of genes across species barrier possible	Restricted to sexually compatible gene pool
	Time-consuming	Results obtained in limited number of generations	Requires many generations
	Transfer of untargeted genes	Transfer of well-defined genes	Potential transfer of multiple (untargeted) genes
Comparative advantages of breeding	Knowledge of metabolic pathways	Requires sufficient knowledge of metabolic pathways	Knowledge of metabolic pathways not required
	Enhanced knowledge of micronutrient metabolism	Limited potential to discover new genes involves	Ability to reveal new genes involved

Abbreviation: GE, genetic engineering.

1 Aspects of both breeding and GE are listed. Note that the importance of these aspects can vary depending on the crop/micronutrient combinations. In some cases, a combined methodology, utilizing both breeding and GE, can be advised.

Ultimately, the challenge for modern agriculture crop development technologies is to combine climate resilient agronomic traits along with nutrition traits as quickly as possible. GE has the potential to achieve this goal within the shortest time frame as compared to other breeding technologies [142]. Unfortunately, overly strict and costly regulations on the application of GE and other advanced technologies is severely constraining potential benefits. There is consensus among scientists around the world that use of GE technologies is safe for humans and the environment [155,156].

Critics of transgenics often identify GE with industrialized, high-input farming approaches, unbeneficial for small farms and harmful to the environment. However, plant breeding in general, and GE in particular, can in some cases significantly lower fertilizer and pesticide use and improve water use efficiency [140]. Higher yields allow for a reduction in the required area to be planted to attain a given food output level, consequently limiting expansion of agricultural land use. GE is compatible with small-scale sustainable farming. If regulatory costs could be reduced, more public and private resources would be devoted to helping small-holder farmers.

## Conclusion

After 20 years of initial investment in biofortification through the HarvestPlus program involving multiple staple food crops and wide collaboration across CGIAR, including efforts by the International Potato Center for provitamin A sweet potato, biofortified crops are being produced and consumed globally in >40 countries. Biofortified crops have been shown to be efficacious in multiple published controlled trials, and biofortification is widely recognized by the international nutrition community as one effective approach among several types of interventions that are needed to reduce mineral and vitamin deficiencies. This is a promising start.

Awareness and efforts to link agriculture and food systems to human nutrition are more evident now than 20 or even 10 years ago, but it is uncertain how this will play out and be sustained. It is important to show successes. However, the impacts of agricultural interventions develop slowly. Biofortification is at the forefront of demonstrating just how resilient, sustainable, and cost-effective agricultural interventions can be for improving nutrition and health.

Although substantial progress has been made, biofortification is not yet tightly woven into the fabric of present-day food systems as part of the core activities of a number of institutions. To continue progress along the same trajectory, it is necessary that sustained funding continues over the longer-term to further develop and improve nutrient densities in plant breeding pipelines. Funding for scale-up/delivery of released biofortified crops in specific countries now can have impacts within 3 to 5 years, so that this type of funding is more easily identified.

To move to a higher trajectory, the impacts of biofortification can be multiplied several-fold through the use of GE and other advanced crop development techniques. Technological change with greater productivity is a natural progression in many sectors. Originally, cell phones were not connected to the internet. In nutrition, multiple micronutrient supplementation is replacing iron-folic acid. Great strides have been made in providing multiple micronutrients through large-scale food fortification. It is in this sense, then, that biofortification is a proven, but “newly emerging” technology. The best is yet to come.

## Author contributions

The authors’ responsibilities were as follows—HB, KL, JJ, BM, RA, MH, WP: designed research; HB, KL, JJ, BM, RA, MH, WP: conducted research; HB, JF, KL, JJ, RR, DVDS, RZ, EB, LRB, BM: wrote the paper; HB: had primary responsibility for final content; and all authors: read and approved the final manuscript.

## Conflict of interest

The authors report no conflicts of interest.

## Funding

RR and RZ report financial support was provided by the Bill & Melinda Gates Foundation. DVDS reports financial support was provided by Ghent University (BOF18-GOA-042). JF, EB, LRB, BM, RA report financial support was provided by HarvestPlus (www.HarvestPlus.org), a global program working to develop and promote biofortified food crops that are rich in vitamins and minerals needed for good health. HarvestPlus’ principal donors are the UK government and the Bill & Melinda Gates Foundation. The views expressed do not necessarily reflect those of HarvestPlus.
