# Supplementary material for: Biofortification: Future Challenges for a Newly Emerging Technology to Improve Nutrition Security Sustainably
Source: Curr Dev Nutr. 2024 Oct 19;8(12):104478. doi: 10.1016/j.cdnut.2024.104478 (PMC11635736; doi:10.1016/j.cdnut.2024.104478)
Supplement: Multimedia component 1 [file mmc1.docx]

**Supplementary Material 1. The Retail Price of Biofortified Staple Foods**

A basic tenet of the biofortification strategy is that biofortified staples will sell for the same price as non-biofortified staple foods. Thus, at no extra cost to household food budgets, biofortified foods may be substituted one-for-one for non-biofortified staple foods. The extra nutrients are free to consumers -- although upfront costs are incurred in agricultural research and initial promotion of biofortified seeds to farmers and biofortified food products to consumers.

The equality of prices is based on the economics of supply and demand. Comparing biofortified with non-biofortified crops, yields are high and production inputs and practices are identical. If initial demand for biofortified foods is high and supplies short, the biofortified price will be higher. However, then profits for production of biofortified crops will be higher (same output and costs, but a higher price and so profits for biofortified crops). This will stimulate more production and supply of the biofortified crop, bringing the price down in equilibrium.

The best example of this price equality is for zinc wheat in Pakistan (see section 4.2.2). Zinc wheat (Akbar 19) is of good quality in terms of consumer characteristics. Flours of similar quality are mixed together in processing. Retail price differentials are determined, not by wheat variety, but by degree of milling and other factors.

[https://www.bing.com/ck/a?!&&p=9aa3dbbb5081dbcaJmltdHM9MTcxODg0MTYwMCZpZ3VpZD0wOTc3N2VkMy1jN2I5LTY3YzktMzZkZC03MWRiYzY2YjY2OWMmaW5zaWQ9NTAwOA&ptn=3&ver=2&hsh=3&fclid=09777ed3-c7b9-67c9-36dd-71dbc66b669c&u=a1aHR0cHM6Ly93aGF0aXNwcmljZS5way9hdHRhLXByaWNlLWluLXBha2lzdGFuLXRvZGF5Lw&ntb=1](https://eur03.safelinks.protection.outlook.com/?url=https%3A%2F%2Fwww.bing.com%2Fck%2Fa%3F!%26%26p%3D9aa3dbbb5081dbcaJmltdHM9MTcxODg0MTYwMCZpZ3VpZD0wOTc3N2VkMy1jN2I5LTY3YzktMzZkZC03MWRiYzY2YjY2OWMmaW5zaWQ9NTAwOA%26ptn%3D3%26ver%3D2%26hsh%3D3%26fclid%3D09777ed3-c7b9-67c9-36dd-71dbc66b669c%26u%3Da1aHR0cHM6Ly93aGF0aXNwcmljZS5way9hdHRhLXByaWNlLWluLXBha2lzdGFuLXRvZGF5Lw%26ntb%3D1&data=05%7C02%7CH.BOUIS%40CGIAR.ORG%7Ce7657a7072cb4206efc208dc91144252%7C6afa0e00fa1440b78a2e22a7f8c357d5%7C0%7C0%7C638544763432278613%7CUnknown%7CTWFpbGZsb3d8eyJWIjoiMC4wLjAwMDAiLCJQIjoiV2luMzIiLCJBTiI6Ik1haWwiLCJXVCI6Mn0%3D%7C0%7C%7C%7C&sdata=D3QvUsoZLWWZiHtkll6jCB3DLbaK39%2BDmRBr9SjOOgE%3D&reserved=0)

Akbar 19 now comprises a high percent of total supply. Markets for Akbar 19 seed are no longer thin. The price can be considered to be in equilibrium. Ever higher wheat productivity relieves pressure on wheat prices in general to rise as demand increases through population growth.

Studies of prices of biofortified foods from first introduction to present have not been undertaken. There is some anecdotal evidence that the biofortified price is higher in the initial stages of introduction. For example, to quote from Funes et al (75):

“Although our community survey collected prices of regular beans, there was anecdotal evidence that the price of HYV-IBB [high-yeilding variety – iron biofortified bean] seeds can reach a premium price up to 20% higher than the price of regular beans or non-IBB seeds.”

Funes et. al. (75) documented a 20-25% increase in biofortified bean yields. Thus, it would be clear why, in short supply, biofortifed beans would bring a premium. However, as supplies of biofortified beans are driven up over time, prices will fall. Rwandan consumers benefit from increased bean productivity apart from the fact that they contain more iron.

**Supplementary Material 2. The Cost of Vitamin A Supplementation (see section 6.2)**

There is no single source of information to derive these numbers on the costs of vitamin A supplementation.

**Number of Capsules Distributed Globally Each Year**

“Nutrition International procures more than 75% of the global vitamin A requirements for programme countries (approximately 460 to 530 million capsules a year), which goes through UNICEF as contributions-in-kind (CIK).” <https://www.unicef.org/supply/media/491/file/vitamin-A-supplementation-market-and-supply-update.pdf>.

**Cost per Capsule**

In a 2007 article on the cost of vitamin A capsule distribution, Neidecker-Gonzales et al. (3) concluded, “Total costs are lowest (roughly US$0.50 per capsule) **in Africa,** where wages and incomes are lowest, US$1 in developing countries in Asia, and US$1.50 in Latin America. Overall, this study derives a much higher global estimate of costs of around US$1 per capsule.”

In the intervening 15 years or so since the above publication appeared, international prices have roughly doubled due to inflation (<https://www.imf.org/external/datamapper/PCPIPCH@WEO/WEOWORLD>).

This dovetails with more recent cost estimates for several **African** countries: “Using the approach described in the section above, we estimate that it costs $1.10 on average to deliver a vitamin A supplement in Helen Keller-supported VAS mass distribution programs. Full details are in [**this spreadsheet**](https://docs.google.com/spreadsheets/d/1wJaWPx1wpseVbqkNYapIdc3V2plHjLy-8RhU78053lg/edit?usp=sharing) .
